# Serological Evidence of *Orthopoxvirus* Circulation Among Equids, Southeast Brazil

**DOI:** 10.3389/fmicb.2018.00402

**Published:** 2018-03-08

**Authors:** Iara A. Borges, Mary G. Reynolds, Andrea M. McCollum, Poliana O. Figueiredo, Lara L. D. Ambrosio, Flavia N. Vieira, Galileu B. Costa, Ana C. D. Matos, Valeria M. de Andrade Almeida, Paulo C. P. Ferreira, Zélia I. P. Lobato, Jenner K. P. dos Reis, Erna G. Kroon, Giliane S. Trindade

**Affiliations:** ^1^Departamento de Microbiologia, Instituto de Ciências Biológicas, Universidade Federal de Minas Gerais, Belo Horizonte, Brazil; ^2^Centers for Disease Control and Prevention (CDC), Atlanta, GA, United States

**Keywords:** *Vaccinia virus*, horsepox, bovine vaccinia, Equine Vaccinia, Horse diseases

## Abstract

Since 1999 *Vaccinia virus* (VACV) outbreaks involving bovines and humans have been reported in Brazil; this zoonosis is known as Bovine Vaccinia (BV) and is mainly an occupational disease of milkers. It was only in 2008 (and then again in 2011 and 2014) however, that VACV was found causing natural infections in Brazilian equids. These reports involved only equids, no infected humans or bovines were identified, and the sources of infections remain unknown up to date. The peculiarities of Equine Vaccinia outbreaks (e.g., absence of human infection), the frequently shared environments, and fomites by equids and bovines in Brazilian farms and the remaining gaps in BV epidemiology incited a question over OPV serological status of equids in Brazil. For this report, sera from 621 equids - representing different species, ages, sexes and locations of origin within Minas Gerais State, southeast Brazil – were examined for the presence of anti-*Orthopoxvirus* (OPV) antibodies. Only 74 of these were sampled during an Equine Vaccinia outbreak, meaning some of these specific animals presented typical lesions of OPV infections. The majority of sera, however, were sampled from animals without typical signs of OPV infection and during the absence of reported Bovine or Equine Vaccinia outbreaks. Results suggest the circulation of VACV among equids of southeast Brazil even prior to the time of the first VACV outbreak in 2008. There is a correlation of OPVs outbreaks among bovines and equids although many gaps remain to our understanding of its nature. The data obtained may even be carefully associated to recent discussion over OPVs history. Moreover, data is available to improve the knowledge and instigate new researches regarding OPVs circulation in Brazil and worldwide.

## Introduction

After 1980, following cessation of mass smallpox immunization, *Vaccinia virus* (VACV) – the *Orthopoxvirus* (OPV) used during the successful World Health Organization Smallpox Eradication Campaign (Fenner and Henderson, 1988) – emerged as a zoonosis in India, Pakistan and Brazil (Essbauer et al., 2010) VACV outbreaks involving dairy cattle and humans were first described in Brazil in 1999 (Damaso et al., 2000). The number of Bovine Vaccinia (BV) reports continues to increase. BV commonly affects milking cows and cattle workers of small properties. Exanthemas are often located at the udder and teats of cows. All stages of the lesions – macule, papule, vesicle, pustule, ulcer, and scab – are highly contagious and direct or indirect contact with an abrasion or bare skin is enough to cause VACV infection in humans. Humans are often infected during manual milking; exanthemas are usually found on their hands and forearms (Damaso et al., 2000; Trindade et al., 2003).

*Vaccinia virus* is currently the only known OPV circulating in Brazil and has recently been detected in other South American countries such as Uruguay, Argentina and Colombia. However, other OPVs are known to occur in the Americas (Emerson et al., 2009; Gallardo-Romero et al., 2012). Recently, various species of rodents have been investigated as possible reservoirs for VACV and the virus has been detected in sylvatic and synantropic species (Abrahão et al., 2009, 2010a).

A VACV outbreak in horses occurred at a breeding center at south Brazil in 2008 and constitutes the first national report of VACV infected horses (Brum et al., 2010; Campos et al., 2011). Surprisingly, no relationship or contact to infected bovines was identified and the source of infection is not known up to date. A second and similar outbreak occurred in southeast Brazil during 2011 (Matos et al., 2013). Horses from different properties developed exanthemas on their muzzles and no infected bovine, human or other animal were determined as the possible source of infection, nor were any non-equine species observed to be infected as a result of the outbreak (Matos et al., 2013). The third outbreak reported the occurrence of oral lesions in donkeys and mules from the northeastern region of the country. Molecular findings indicated a VACV from Group 1 as the etiological agent and again no other species but *Equus sp*. have been connected to this outbreak (Abrahão et al., 2017).

Brazil has the largest herd of horses in Latin America and the third in the world. Together with the mules and donkeys are 8 million head, moving R$ 7.3 billion, only with the production of horses. Brazil is the eighth largest exporter of equine meat but when it comes to the export of live horses, the numbers are significant and expanded by 524% between 1997 and 2009. The largest Brazilian population of horses is in the Southeast, followed by the Northeast, Midwest, South and North, with one of its main functions being the daily work in agricultural activities, where about five million animals are primarily used for the management of cattle (MAPA).

To better comprehend the relationship between VACV and equids in Brazil, serum from more than six hundred equids from all mesoregions of Minas Gerais (MG) state, southeast Brazil, were analyzed for evidence of OPV exposure. Previous exposure of equids to OPV was evaluated by two different assays: the plaque reduction neutralization test (PRNT) to identify neutralizing antibodies (gold standard), and an enzyme-linked immunosorbent assay (ELISA). This latter method allows for assessment of non- neutralizing anti-OPV IgG antibodies.

This study provides an evaluation of serological evidence of VACV exposure in equids, expanding the epidemiological hypothesis of VACV circulation in Brazil, and potentially in other countries where VACV has been reported but its natural cycle is not well understood. The introduction of VACV into the Americas and its circulation among European horses prior to the 19th century have gained greater attention recently and highlighted the importance of horses in OPVs history (Damaso, 2017; Esparza et al., 2017; Schrick et al., 2017). Insights of the possible correlation of OPVs outbreaks among cows and equids have been evaluated and more data is currently available to improve the knowledge and also the development of control and prevention methods of OPVs circulation worldwide, specially VACV-like viruses.

## Materials and Methods

### Samples

**FIGURE 1:**
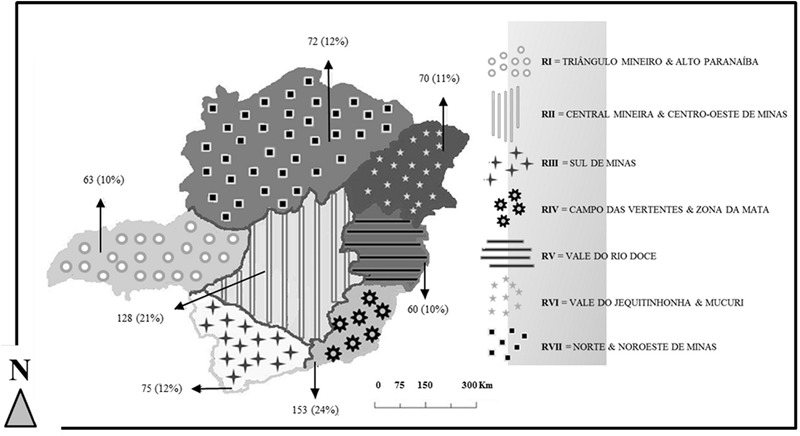
Mesoregions of Minas Gerais State and equine specimens collected. The absolute number and correspondent percentage of serological specimens [n(%)] from each mesoregion is indicated by the arrows.

A total of 621 sera were examined from seven mesoregions of MG State. The mesoregions are defined by the animal defense bureau of the state of MG. **Figure 1** describes the abbreviations for regions that are used throughout this report. Four hundred seventy-eight sera from equids (*Equus caballus, E. asinus* and hybrids) were collected from numerous locations around MG state during the second half of 2003 and first half of 2004 (**Figure 1**). An additional 74 sera of *E. caballus* from RIV (**Figure 1**) were collected in July 2011, from the second known episode of Equid Vaccinia (EV) in Brazil. The serum specimens were collected approximately 1 month after the onset of symptoms, and clinical diagnoses were confirmed using molecular assays (Matos et al., 2013). The last 69 sera were randomly collected in RII during the second half of 2012 (**Figure 1**). All specimens were donated by the “Departamento de Medicina Veterinaria Preventiva, Escola de Veterinaria, Universidade Federal de Minas Gerais”. All clinical specimens were derived from domestic equids on private properties and were collected by a veterinarian according to standard sanitary protocols in agreement to the requirements of national and local livestock agencies, “Ministerio da Agricultura, Pecuaria e Abastecimento” and “Instituto Mineiro de Agropecuaria”, respectively. The sampling procedure was submitted and approved by the “Comite de Etica em Experimenação Animal (CETEA)” in accordance to the requirements of animal research of Universidade Federal de Minas Gerais (UFMG), MG state, Brazil (protocol number 131/2010, approval date 8/2010 and expiration date 8/2015).

Sterile vacuum blood collection tubes and needles were used to collect approximately 5 mL of blood from each animal while the animal was restrained with a bridle. The area of the jugular vein was cleaned with cotton soaked in 70% alcohol, and samples were collected from the jugular vein. A sterile and dry piece of cotton was used to apply pressure to the sampled area.

The name of the responsible employee and/or owner and the addresses of all properties were collected. Permission for sampling the horses was verbally granted. There was a verbal agreement to maintain confidentiality of the names and specific geographic location of each property. Coordinates from the regions further mentioned in this study are as follows (Datum: WGS84): RI or TRIANGULO MINEIRO AND ALTO PARANAIBA 19°16′18.45″South and 48°20′59.95″West; RII or CENTRAL MINEIRA AND CENTRO-OESTE DE MINAS 18°51′15.52″South and 44°29′22.88″West; RIII or SUL DE MINAS 21°19′18.57″South and 45°48′09.67″West; RIV or CAMPO DAS VERTENTES AND ZONA DA MATA 21°30′01.91″South AND 43°27′57.24″West; RV or VALE DO RIO DOCE 19°28′22.44″South AND 41°46′46.62″ West; RVI or VALE DO JEQUITINHONHA AND MUCURI 16°58′31.86”South and 41°15′41.43″West; RVII or NORTE E NOROESTE DE MINAS 16°17′40.87″South and 44°41′03.21″West.

### Epidemiologic and Demographic Variables

Four hundred seventy-eight sera were collected for multiple research purposes and there is no record of clinical signs suggestive of OPV acute infection among their database, as well as for the 69 sera randomly collected in RII. All these 547 sera have been submitted to PCR to OPV vgf gene according to Abrahão et al. (2010b) to detect a possible silent DNAmia; none trialed positive (data not shown). The geographic location, species, age, and sex of each equid sampled are also recorded.

The 74 sera (from the second EV outbreak notified in Brazil) were collected from equids with and without OPV-like exanthemas as described by Matos et al. (2013). According to them, viruses were isolated from sampled lesions and DNAmia was detected among several horses approximately 1 month after the first infected equid was noticed. Authors demonstrated through molecular methods a VACV sample to be the etiologic agent of this outbreak. The geographic location, species and sex of each equid sampled are recorded.

The location data for equids was aggregated by state subdivision. Subdivisions (mesoregions) are shown in **Figure 1** along with the absolute number and corresponding percentage of equids evaluated from each area. Since BV has traditionally been associated with dairy production in Brazil, it is helpful to consider dairy production activities in MG in relation to equid sampling locations. Milk producing properties are found throughout MG but several distinctions are found between the different mesoregions. Small dairy properties are concentrated at RIV and RII, where only one municipality had properties with areas larger than or equal to 69,106 Km^2^. RIV is the area where the second known case of Brazilian EV occurred (Matos et al., 2013). Although traditionally known for its pure breed beef cattle herds, RI produced between 9 and 100 million of liters of milk during the year of 2006, the highest values found in MG State during the last census. RI properties also occupy the largest areas related to all other mesoregions as nearly 50% of its municipalities have properties with areas^1^ over 69,106 Km^2^.

### Serological Assays

#### Plaque Reduction Neutralization Test

The PRNT was performed according to (Newman et al., 2003). Briefly, six well plates with BSC-40 cell monolayers (ATCC^®^ CRL-2761) were inoculated with a 2.5% serum solution plus 150 PFU of VACV Western Reserve (WR) per well. Before infection, sera/WR solutions were incubated overnight at 37^o^C. To maintain the viability of the virus control, fetal bovine serum (FBS) was added to this solution at the same concentration (2.5%). The cell control contained 2.5% FBS media only. After infection, 1 h of adsorption was followed by the addition of 2 ml of 1% FBS media per well and incubation of all monolayers at 37^o^C and 5% CO_2_ for approximately 48 h. After typical VACV-WR cytopathic effects were clearly observed, all monolayers were fixed with 3.7% formaldehyde and stained with 1% crystal violet.

All samples were tested in triplicate and the number of PFU in each well was enumerated. Positive sera (positive for neutralizing antibodies) were defined as those samples that had PFU below the 50% PFU of the viral control.

#### IgG ELISA

Flat-bottom 96 well plates (Nunc MaxiSorp^®^) were coated with inactivated and purified VACV- WR viral particles; these plates were treated with a solution of PBS plus tween 20 (0.05%) and 5% casein. Sera were diluted 100x in a solution of PBS plus tween 20 (0.05%) and 1% casein.

To detect antibodies, anti-horse IgG conjugated to horse radish peroxidase (HRP) (SIGMA-ALDRICH^®^) and tetramethylbenzidine (TMB, BD^®^) were used for colorimetric detection at the concentration of 1:20000. All samples were tested in duplicates, and the results were read at 450 nm. Five known negative equid control sera were used. The average of all five negative controls minus three times their standard deviation determined the cutoff value for each plate.

Additionally, a known positive equid serum was tested for each plate as a positive control.

### Statistical Analysis

Epidemiologic and demographic variables as well as qualitative laboratory findings were analyzed using parametric and non-parametric statistical tests. Pearson Chi-square test was employed for analyzing multiple-category non-parametric data. The Mantel-Haenszel common odds ratio (OR) and Fisher’s exact tests (2-tailed) were used for categorical variables. A *p*-value < 0.050 was used to assign significance of association. All analyses were performed using IBM^®^ SPSS Statistics version 19.

## Results

**FIGURE 2:**
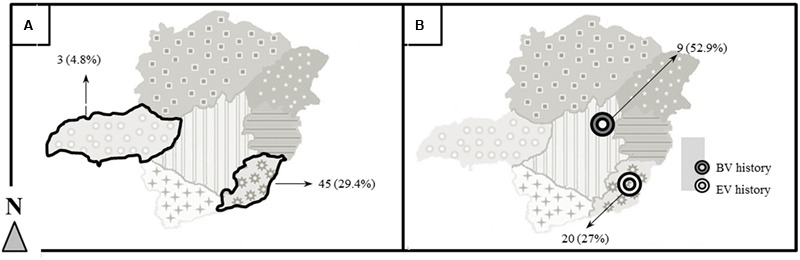
Regional differences in seropositivity and a history of EV and BV outbreaks. **(A)** Two mesoregions of MG presented a discrepancy in equid seropositivity (RI and RIV). Both are highlighted; their absolute number and correspondent percentage of seropositive equids are indicated by arrows [n(%)]. **(B)** A difference of OPV seropositivity among equids derived from areas with EV or BV history was also observed. The white target indicates an area with a history of EV; the black target indicates an area with a history of BV. The absolute number and correspondent percentage of seropositive are indicated by arrows [n(%)].

A total of 128 equids (20.6%) were seropositive for OPV by either the ELISA or PRNT assay (**Table 1**). Seropositive animals were found in all mesoregions of MG state. There was a lower frequency of seropositive equines in RI (4.8%, western MG) and RVII (13.9%, northern MG) and a higher frequency in RIV (29.4%, southeast) compared to the other regions of MG. The results from RI and RIV significantly deviate (-16% and +9%, respectively) from that which would be expected by chance alone (Chi-square 20.5, df =6, 2-sided *p* = 0.002). Moreover, a statistically significant difference was observed between these two regions (**Figure 2A**).

**Table 1:** OPV seropositivity of equids by mesoregion.

Mesoregions	OPV antibody positive^∗^[n(%)]	Total
RI	3 (4.8)^†^	63
RII	27 (21.1)	128
RIII	19 (25.3)	75
RIV	45 (29.4)^†^	153
RV	12 (20)	60
RVI	12 (17.1)	70
RVII	10 (13.9)	72
**Total**	**128 (20.6)**	**621**

*^∗^positive by either PRNT or ELISA. ^†^ > 8% deviation from that expected by chance alone.*

The location of the equids was significantly associated with seropositivity (Chi-square 20.5, df = 6, 2-sided *p* = 0.002). No significant relationship was observed for species, age or sex. The existence of seropositive equids throughout MG State, represents a unique result, since the possibility of VACV natural circulation among horses had been hypothesized, but not demonstrated.

Except in RII and RIV, a history of prior BV or EV occurrence was determined by property owner report only; such reports could not be independently confirmed. In cases where the property owner was not aware of the disease, pictoral representations of typical VACV lesions of humans, bovines and equids were provided. Regarding animals from properties whose owners confirmed BV or EV history – specific cases where samples were sent by IMA to official laboratories and results confirmed VACV infection – the frequency of seropositive equids from BV areas was almost two times higher (52.9%) than the frequency of seropositive equids in EV areas (27%). Furthermore, seropositive equids were found to be three times more likely to be from a property which formerly experiences a BV episode than those from areas where EV has occurred (OR = 3.0, 95% CI, 1.03–8.96, *p* = 0.044) (**Table 2** and **Figure 2B**).

**Table 2:** OPV seropositivity of equids on properties with a history of EV or BV.

		OPV antibody positive^∗^			
			Negative	Positive	Total
**Epidemiological history**	**EV history**	N	54	20	74
		%	73.0%	27.0%	100.0%
	**BV history**	N	8	9	17
		%	47.1%	52.9%	100.0%

**Total**		N	62	29	91
		%	68.1%	31.9%	100.0%

*^∗^positive by either PRNT or ELISA.*

## Discussion

These results demonstrate OPV exposure among equids in MG state, Brazil. As no OPV other than VACV has been proven to circulate in the country, results are hypothesized to be most likely related to the circulation of VACV. Moreover, the presence of serologically positive equids from 2003 to 2004 demonstrates that exposures, and perhaps unrecognized infections, occurred prior to the first described outbreak in Brazilian equids in 2008 (Brum et al., 2010). Results regarding the lower seropositivity observed in RI and higher seropositivity in RIV corroborate several studies in bovines, which associate intense VACV circulation with small property size (Kroon et al., 2011). Also, this result would be expected if there is a relationship between VACV circulation among equids and bovines. RIV is an important dairy region where several BV outbreaks have been reported (Lobato et al., 2005; Trindade et al., 2006; De Souza Trindade et al., 2007). The states that share a boundary with RIV – Espirito Santo and Rio de Janeiro – are also known for the frequent occurrence of BV (Kroon et al., 2011). RI, on the other hand, is known for its large herds of beef cattle^2^ and fewer BV reports (Kroon et al., 2011).

When equids and bovines are maintained together on a single property, their degree of contact is often dictated by the size of the operation and type of production that occurs on the property. In small dairy herds, because of the nature of the work required, equid contact with bovines is considerably more intense than on larger, more sophisticated dairy farms. During dry seasons, these semi-intensive small properties require daily feed dispersal to support dairy production. The transportation of feed on these properties is usually accomplished by equids. Cattle are herded towards the corral and later back to the pastures, often by a human riding an equid. It is important to point out that despite its use in several other activities as sports, leisure and even therapy, one of its main functions, however, remains the daily work in agricultural activities, where about five million animals in Brazil are used primarily for the MAPA. In these situations, it is common for equids to freely access corral areas, share pastures, water, and feeding devices with bovines; therefore, equids housed on small properties with dairy cattle may indeed be more exposed to any pathogen circulating among the dairy cattle as compared to equids involved with beef cattle production or that are found on more technologically sophisticated farms.

The pure breed beef cattle that predominate in RI are highly valued economically^3^. This additional capital value allows for broader investments in a given property’s dairy production. Therefore, the lower seropositivity observed among equids from this region may be a reflection of the investment-based technological innovations that have proliferated throughout the region. Increasing levels of sophistication on dairy properties may lead to the substitution of horses for tractors and crawlers thereby reducing bovine and equid contact. When equids from areas with a previous occurrence of BV versus a previous occurrence of EV were evaluated, a higher number of seropositive equids were found to be from BV properties.

Evaluating the typical systems in which horses are kept, opportunities for disease acquisition and transmission are somewhat different from those typical for small dairy cattle properties. Properties which dedicate part of their labor force to breeding horses tend to have individual stalls for their stallions; pastures present a density of equids considerably lower than those used with dairy cattle. Even if VACV replication and shedding is naturally efficient among horses and bovines, equids exposure would be significantly lower considering typical management practices for horses – those that are not working animals on a dairy farm.

The demonstration of VACV shedding on feces from deliberately infected cows (Rivetti et al., 2013) and the seroconversion of naïve animals after direct or indirect contact with this material (D’Anunciação et al., 2012) corroborate the possible co-dependency of VACV circulation observed among equids and bovines, as equids exposed to an OPV positive herd and environment may induce these equids seroconversion. It has been suggested that VACV is more successful in replicating and being shed by bovines as compared to equids; the experimental infection in equids with Brazilian samples of VACV indicated a low level of replication at the inoculation site with mild cutaneous lesions when compared with the course of infection of other hosts. The authors hypothesized equids have a low potential for viral maintenance and transmission to other species, albeit being susceptible to VACV infection (Barbosa et al., 2016).

Damaso, Esparza, Schrick, and collegues have revisited Edward Jenner’ Inquiry in 2017 and vaccines tested by the doctor with specimens obtained from horses have come to all attention. Jenner attested “horse material” would most likely protect humans against smallpox only if previously inoculated in cows, as it rarely produced the “take” (exanthema developed at the site of inoculation) when directly sampled from horses and promptly inoculated in humans (reviewed by Baxby, 1999). Considering the possibility of VACV-like samples circulation in 19th Europe and their unknown use by Jenner, these observations may relate to what Barbosa called “a low level of replication” of Brazilian VACV samples in deliberately infected equids. The absence of human cases associated to all EV outbreaks reported in Brazil so far, also follows the pattern previously proposed and instigate the conclusion of a probable route of VACV from bovines to equids, as these last apparently are less effective in shedding the virus. Many characteristics of the infection of equids by VACV, however, remain to be investigated and assumptions should not be made based only at these studies.

Most importantly the evidence of silent (or possibly unreported) VACV exposure and disease in Brazilian equids in southeast Brazil has been demonstrated. VACV multiplication in equids occurs and even if it is less effective than in bovines, virus shedding into the environment constitutes a potential source of infection to other animals, domestic or wild, and to humans. Little is known about possible VACV reservoirs in Brazil as well as the exact importance of equids for VACV maintenance and circulation. Care must be taken in order to avoid VACV dissemination; veterinarians, horse and cattle caretakers, and all others involved in the equid industry should be informed of the risks related to EV.

Little investment has been made so far to prevent and control VACV infections in Brazil, either associated to BV or to the relatively recent EV. Disease prevention efforts are typically intensified only after a county suffers a significant outbreak. Municipalities that are potentially at risk remain uninformed about the disease until the impacts are apparent, including reduced milk production and painful human infections during BV outbreaks, bovine or equine morbidity during BV and EV outbreaks, respectively. Livestock disease surveillance programs have a role in outbreaks with suspected VACV cases, concomitant notifications to human public health authorities would speed the rapid inception of control measures, but research constitutes still the major investment related to VACV in Brazil. The continuation of serological monitoring and complementary research of VACV in equids is therefore fundamental to assess the spectrum of VACV impacts throughout the country, as well as to the development of prevention and control methods for its circulation nationwide.

## Author Contributions

IB did the standardization of the laboratorial techniques, field and laboratory work, data compilation, and paper writing. MR and AMM were responsible for guidance, statistical analysis, and paper writing. POF and FV did the field and laboratory work. LA, GC, ACM, VAA, ZL, and JR did the field work. PCF, ZL, JR, and EK provided guidance. GT contributed to the idealization of the project, guidance, and paper writing.

## Conflict of Interest Statement

The authors declare that the research was conducted in the absence of any commercial or financial relationships that could be construed as a potential conflict of interest.

## Footnotes

1 http://www.ibge.gov.br/

2 http://www.abcz.org.br/

3 http://www.abcz.org.br/
